# A diagnostic challenge of tongue botryomycosis miming mycetoma—A case report

**DOI:** 10.1002/ski2.433

**Published:** 2024-08-05

**Authors:** Emmanuel Edwar Siddig, Ayman Ahmed

**Affiliations:** ^1^ Department of Medical Microbiology and Infectious Diseases Erasmus MC University Medical Center Rotterdam Rotterdam the Netherlands; ^2^ Faculty of Medical Laboratory Sciences University of Khartoum Khartoum Sudan; ^3^ Institute of Endemic Diseases University of Khartoum Khartoum Sudan; ^4^ Swiss Tropical and Public Health Institute (Swiss TPH) Allschwil Switzerland; ^5^ Faculty of Science University of Basel Basel Switzerland

## Abstract

Botryomycosis of the tongue is a rare chronic bacterial infection that presents as nodular masses, mimicking other infectious or neoplastic conditions such as mycetoma. A case of an 80‐year‐old male was presented with painless swelling on the right lateral side of his tongue to the outpatient clinic. Biopsy and microbiological investigations revealed an unexpected *Staphylococcus aureus*‐related botryomycosis. This case highlights the diagnostic challenge for unusual clinical presentations of bacterial infections. Healthcare providers in countries endemic with diseases that manifest similarly should investigate thoroughly to ensure a positive clinical outcome through early diagnosis and effective case management.

## Graphical abstract

Botryomycosis, an infrequent bacterial infection, was unexpectedly identified as the aetiology behind the patient's tongue lesion. Botryomycosis mimics mycetoma clinically, which is common among infectious diseases. Therefore, healthcare providers should be more exhaustive during the differential diagnosis. Countries endemic with several diseases that have overlapping symptoms and clinical presentations, should invest more on improving the diagnostic capacity.

## 1 BACKGROUND

Botryomycosis is classified as a rare granulomatous disease of bacterial origin, presenting in two forms: cutaneous and visceral.^1^ The subcutaneous form often bears clinical resemblance to actinomycetoma.^2^ Histological diagnosis involves identifying eosinophilic subcutaneous regions enclosing densely packed, dark blue bacterial colonies. The lung is the most commonly affected site in the visceral form, often occurring in conjunction with chronic conditions such as cystic fibrosis.^3^

In Sudan, several risk factors are intensifying the spread of endemic diseases and emergence of novel infections. These factors include climate change and war‐enhanced displacement and dynamic of human and animal populations.^4^, ^5^, ^6^ Furthermore, due to the limited diagnostic capacity, misdiagnosis and delay in detecting co‐infection are commonly occurring.^7^, ^8^, ^9^ Here, we present a case of a male patient from Sudan exhibiting botryomycosis on the right lateral side of his tongue, which clinically mimics mycetoma.

## 2 CASE PRESENTATION

**FIGURE 1:**
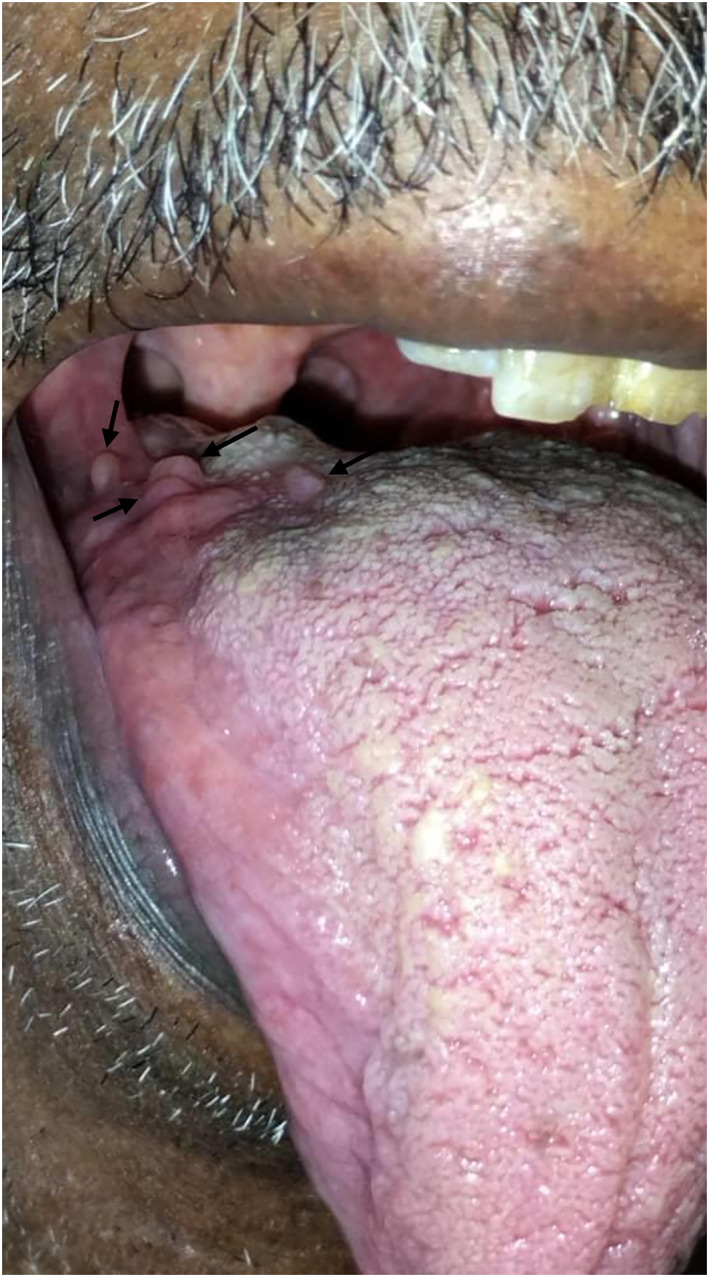
Depicts the clinical appearance of the patient, revealing the presence of raised subcutaneous nodules, measuring 1 cm in diameter, on the right lateral aspect of the tongue, which are mildly tender to touch.

An 80‐year‐old male from Al Geziera state, central Sudan, presented with a soft irregular painless swelling measuring 1 cm in diameter on the right lateral side of his tongue (Figure 1). The swelling had been present for 17 months, with gradual onset and discharge of white materials. The patient has a history of heavy smoking, but no history of mycetoma infection in the family. He was negative for human immunodeficiency (HIV), had no history of organ transplantation, nor other pre‐existing medical conditions. However, due to the morphological mimicry, the patient was initially suspected for mycetoma: therefore, screening for the causative agents of mycetoma was prompted but they were all negative. Later, he recalled a history of trauma that involves biting his tongue during chewing.

## 3 INVESTIGATIONS

**FIGURE 2:**
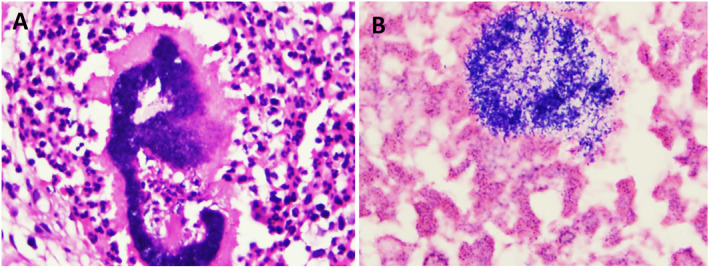
(a) A microphotograph of a histological section reveals an irregularly shaped grain surrounded by inflammatory cells, appearing blue with haematoxylin and eosin (H&E) staining. The bacterial colonies are encased in the granulation tissue showcasing prominent histiocytic and mixed inflammatory cellular infiltrates. Additionally, a Splendore–Hoeppli reaction is observed at the periphery of the section, displaying a light red stain (H&E 400X). (b) A gram‐stained smear displays colonies characterised by gram‐positive cocci staining pattern.

Viral screening for HIV and Hepatitis B and C viruses were negative. Liver function tests showed that serum bilirubin was 0.42 mg/dL, total protein was 7.6 g/dL, serum albumin was 5.8 g/dL, alkaline phosphatase was 78 U/L, aspartate aminotransferase was 23 U/L and alanine aminotransferase was 30 U/L. Renal function test showed a normal value of urea in blood (33 mg/dL) and serum creatinine (0.68 mg/dL). Complete blood count examination showed that leucocytosis was 11.0 × 10^3^, haemoglobin was 12.8 g/dL and platelets count was 376 × 10^3^. A deep excisional biopsy taken at a private dental clinic showed bacteria surrounded by an eosinophilic matrix with club‐like projections, consistent with a Splendore–Hoeppli phenomenon (Figure 2a). The morphology of the grains was different from typical infections with *Staphylococcus somaliensis*, *A. madurae* and *A. pelletieri*. Gram stain and culture revealed the presence of gram‐positive cocci (Figure 2b), and later it was confirmed as *Staphylococcus aureus*.

## 4 DIFFERENTIAL DIAGNOSIS

In evaluating a painless swelling measuring 1 cm on the right lateral side of the tongue suspected to be botryomycosis, it is crucial to consider a broad range of differential diagnoses. These may include benign conditions such as fibroma, mucocele, and oral lymphoepithelial cysts, as well as potentially malignant entities such as squamous cell carcinoma, salivary gland tumours and lymphomas (Table 1). Furthermore, infectious aetiologies such as tuberculosis, syphilis and fungal infections should be ruled out (Table 1). A thorough clinical examination coupled with diagnostic investigations such as biopsy would be instrumental in differentiating botryomycosis from these various possibilities.

**TABLE 1:** Differential diagnosis of tongue swelling.

Infection	Abscess
	Actinomycetoma
	Botryomycosis
	Inflammatory granuloma due to tuberculosis
	Granuloma due to syphilis
	Leishmaniasis
Neoplasia	Neuroma
	Haemangioma
	Lymphangioma
	Carcinoma
	Sarcoma

## 5 TREATMENT

As soon as we made the final diagnosis, we started the patient on amoxicillin (1000 mg twice daily for 14 days) and they have fully recovered.

## 6 OUTCOME AND FOLLOW UP

Inspecting the patient's mouth showed full recovery, with no evidence of residual lesions observed.

## 7 DISCUSSION

This case highlights the diagnostic challenge that can arise when encountering uncommon clinical manifestation of bacterial infection such as unusual lesions of the tongue. The patient's history and examinations have initially raised the possibility of either infectious or neoplastic aetiologies, with a wide differential diagnosis with mycetoma on the top of the list (Table 1). However, the subsequent biopsy results and microbiological investigations helped reaching an unexpected final diagnosis of *S. aureus*‐related botryomycosis.

Botryomycosis is an unusual, long‐term bacterial infection that can affect different areas of the body, with oral involvement being relatively uncommon.^1^ The development of botryomycosis is associated with various risk factors, including alcoholism, diabetes mellitus, HIV infection and physical trauma.^1^, ^2^ The condition is more commonly observed in individuals with weak immune systems.^2^

The condition is characterised by the development of nodular masses on the tongue in our case, which can often be mistaken for other infectious or tumour‐like conditions, such as mycetoma. It is essential to differentiate botryomycosis from other conditions to facilitate timely intervention and effective case management.^1^

The use of a deep excisional biopsy and microbiological investigations were essential in identifying the causative agent and initiating effective treatment.^1^, ^2^ The case also emphasises the importance of pursuing alternative possibilities during the differential diagnosis and considering atypical presentations of diseases. Reports about unusual clinical manifestations of infections are increasingly growing.^3^, ^7^ Therefore, to improve healthcare and clinical outcomes of patients, disease control programs in endemic countries should invest in building diagnostic capacity including infrastructure, equipment and training the healthcare providers as well as integrating the use of advanced molecular and genomic tools in the diagnostic services.^7^, ^10^ Despite the cost associated with such capacity building, this will be cost‐effective by reducing the extensive cost of care based on misdiagnosis. Additionally, it will save more lives and reduce the disability‐adjusted life years.^11^

In conclusion, the presentation of an unusual tongue lesion should prompt a thorough diagnostic investigation to avoid misdiagnosis, inappropriate treatment and severe complications. This case indicates that botryomycosis, although rare, can present as a tongue lesion and should be considered in the differential diagnosis of nodular lesions of the tongue, particularly in patients with a history of trauma or infection.

## 8 PATIENT'S PERSPECTIVE

The appearance of the lesion triggered feelings of worry and doubt within me. I found myself becoming more anxious about what the swelling might signify for my health, as well as the steps needed for diagnosis and treatment. This scenario led me to confront a mix of emotions, such as fear, nervousness and a deep need for clear information and comfort. Over time, I developed a serious concern and stress about social stigma and discrimination in my community. I believe that compassionate and informative communication from healthcare providers were major key in easing my fears and helping me feel more empowered as I navigate the journey towards understanding and addressing my condition.

## CONFLICT OF INTEREST STATEMENT

None to declare.

## AUTHOR CONTRIBUTIONS

**Emmanuel Edwar Siddig**: Conceptualization (equal); data curation (equal); formal analysis (equal); investigation (equal); methodology (equal); resources (equal); supervision (equal); validation (equal); visualization (equal); writing – original draft (equal); writing – review & editing (equal). **Ayman Ahmed**: Conceptualization (equal); data curation (equal); formal analysis (equal); investigation (equal); methodology (equal); resources (equal); software (equal); supervision (equal); validation (equal); visualization (equal); writing – original draft (equal); writing – review & editing (equal).

## ETHICS STATEMENT

Written informed consent to publish history, findings and images for educational purposes were obtained from the patient and the study was approved by the University of Khartoum, faculty of medical laboratory sciences committee.

## PATIENT CONSENT

Written informed consent to publish history, findings and images for educational purposes were obtained from the patient.

## Data Availability

The datasets used and/or analysed during the current study are available from the corresponding author on reasonable request.
